# Addressing inaccuracies in BLOSUM computation improves homology search performance

**DOI:** 10.1186/s12859-016-1060-3

**Published:** 2016-04-27

**Authors:** Martin Hess, Frank Keul, Michael Goesele, Kay Hamacher

**Affiliations:** Graphics, Capture and Massively Parallel Computing, Department of Computer Science, Technische Universität Darmstadt, Rundeturmstraße 12, Darmstadt, 64283 Germany; Computational Biology and Simulation, Department of Biology, Technische Universität Darmstadt, Schnittspahnstraße 2, Darmstadt, 64287 Germany

**Keywords:** Substitution matrix, Homologous sequence search, BLOSUM, Correction, RBLOSUM, CorBLOSUM, Performance evaluation, ASTRAL, BLOCKS 13+, BLOCKS 14.3

## Abstract

**Background:**

BLOSUM matrices belong to the most commonly used substitution matrix series for protein homology search and sequence alignments since their publication in 1992. In 2008, Styczynski et al. discovered miscalculations in the clustering step of the matrix computation. Still, the RBLOSUM64 matrix based on the corrected BLOSUM code was reported to perform worse at a statistically significant level than the BLOSUM62.

Here, we present a further correction of the (R)BLOSUM code and provide a thorough performance analysis of BLOSUM-, RBLOSUM- and the newly derived CorBLOSUM-type matrices. Thereby, we assess homology search performance of these matrix-types derived from three different BLOCKS databases on all versions of the ASTRAL20, ASTRAL40 and ASTRAL70 subsets resulting in 51 different benchmarks in total. Our analysis is focused on two of the most popular BLOSUM matrices — BLOSUM50 and BLOSUM62.

**Results:**

Our study shows that fixing small errors in the BLOSUM code results in substantially different substitution matrices with a beneficial influence on homology search performance when compared to the original matrices. The CorBLOSUM matrices introduced here performed at least as good as their BLOSUM counterparts in ∼75 *%* of all test cases. On up-to-date ASTRAL databases BLOSUM matrices were even outperformed by CorBLOSUM matrices in more than 86 *%* of the times. In contrast to the study by Styczynski et al., the tested RBLOSUM matrices also outperformed the corresponding BLOSUM matrices in most of the cases. Comparing the CorBLOSUM with the RBLOSUM matrices revealed no general performance advantages for either on older ASTRAL releases. On up-to-date ASTRAL databases however CorBLOSUM matrices performed better than their RBLOSUM counterparts in ∼74 *%* of the test cases.

**Conclusions:**

Our results imply that CorBLOSUM type matrices outperform the BLOSUM matrices on a statistically significant level in most of the cases, especially on up-to-date databases such as ASTRAL ≥2.01. Additionally, CorBLOSUM matrices are closer to those originally intended by Henikoff and Henikoff on a conceptual level. Hence, we encourage the usage of CorBLOSUM over (R)BLOSUM matrices for the task of homology search.

**Electronic supplementary material:**

The online version of this article (doi:10.1186/s12859-016-1060-3) contains supplementary material, which is available to authorized users.

## Background

One of the most basic tasks in bioinformatics is the search for homologous protein sequences, e.g. to classify newly discovered proteins or to analyze evolutionary relationships. Here, the elementary step is the computation of sequence similarity of any two sequences by so called pairwise alignments using algorithms like Needleman-Wunsch [1], Hirschberg’s algorithm [2], Smith-Waterman [3], and Gotoh’s algorithm [4]. All of these algorithms use substitution matrices to model evolutionary substitution events and gap penalty models to represent evolutionary insertion/deletion events.

The selection of the parameters in these models is a non-trivial task and an important step in homology search [5–7] and phylogeny [8, 9]. Over the years many different substitution matrices have been developed using different techniques such as Markov chain models (PAM) [10], maximum likelihood estimation (VTML) [11] or direct derivation from highly conserved amino acid blocks (BLOSUM) [12].

In order to assess the performance of substitution matrices the state of the art approach applies homologous sequence search on a standardized database with known sequence relations [13, 14]. Here, the ASTRAL database [14, 15] — a subset of the SCOPe database [16, 17] — serves as a gold standard for this benchmark [6, 13, 18–20]. Typically, all sequences of the ASTRAL database are searched against the entire database to obtain a list of found homologs given a set of search parameters.

A well established method to measure the performance of these parameters is the coverage measure at a given errors per query (epq) [13]. In this context, the coverage is similar to receiver operator characteristics (ROC) and uses SCOPe sequence superfamily annotations to classify found homologs as true and false positives. In order to compensate for different superfamily sizes quadratic normalization of the coverage can be applied [6]. As the coverage is strongly depending on the composition of the search database, significance of the results can be estimated via Concerted Bayesian bootstrapping [18].

A frequently used reference for benchmarking are the BLOSUM matrices as these are standard parameters for database search programs such as NCBI BLAST [21] and SSEARCH [22]. While the BLOSUM matrix series was introduced over two decades ago by Henikoff et al. [12], previous work [19] revealed inconsistencies in the cluster weighting procedure of the matrix calculation. Interestingly, correcting these miscalculations did not improve the search performance of the corrected BLOSUM variant (RBLOSUM) for the best benchmark at that time (ASTRAL40 1.69).

Recently, Song et al. [20] presented another approach to address inaccuracies in the BLOSUM matrices by finding optimal unified eigenvectors. Nonetheless, for homologous sequence search, their PBLOSUM matrix was reported to perform consistently worse than BLOSUM62. Thus, BLOSUM serves as an upper bound on the search performance.

The ever improving coverage of the protein sequence space, allowed us to conduct a more detailed analysis of the RBLOSUM correction [19] and its impact on homology search performance. Based on the corrections presented by Styczynski et al. [19] we modified the original BLOSUM code [23] and noticed an additional inaccuracy (corrected code shown in Additional file 1).

The coding problem affects cluster memberships of sequences and necessitates modifications to both the original BLOSUM and the RBLOSUM variant. In short, the published code contains an inaccurate integer based thresholding, so that sequences may be assigned to a particular cluster, even though they do not meet the user-specified clustering threshold. While — on the surface — the induced inaccuracies appear to be minuscule, the resulting substitution matrix entries are *systematically biased* away from the actual conservation tendency intended by Henikoff et al. [12].

The following example illustrates this effect. At a block length of 93 amino acids, a minimum sequence similarity of 62 *%* — corresponding to the threshold used to generate the BLOSUM62 substitution matrix — leads to a similarity threshold of 57.66 identical residues. Or in other words, at least 57.66 identical amino acids between two sequences are required to form a cluster. In the original implementation, this value is truncated to 57 identical residues. In fact, this corresponds to an effective clustering value of just 61.29 *%* which was not intended by the user and may result in mistakenly clustered sequences. A correction of this error in combination with the problems reported earlier [19] prompted us to derive a new substitution matrix series, named CorBLOSUM hereafter. A detailed description of this inaccuracy and an analysis of its impact is discussed in Additional file 1.

In this paper, we analyze the influence of the above mentioned error corrections on the resulting matrices derived from different BLOCKS database compositions in combination with their respective homologous sequence search performance. We present an exhaustive analysis on all available ASTRAL releases at different maximal sequence identities. Hence, our analysis covers 51 test databases in total and is to our knowledge the largest assessment of BLOSUM-type matrix performance to date.

We show that fixing a small coding error results in substantially different CorBLOSUM matrices which beneficially influence homology search performance in comparison to the original matrix. In particular, these new matrices outperform their BLOSUM counterparts in ∼75 *%* of all tested scenarios, especially on recent test databases (ASTRAL versions ≥2.01).

## Method

### Substitution matrices

We calculated the above introduced, different variants of the BLOSUM matrix (BLOSUM, RBLOSUM and CorBLOSUM) using the algorithms described in [12, 19] and the aforementioned CorBLOSUM algorithm (see Additional file 1).

As the magnitude of both error corrections is influenced by the database composition and as newer BLOCKS releases are reported to produce better performing matrices [6], we derived matrices from three different databases: BLOCKS 5, BLOCKS 13+ and BLOCKS 14.3.

The BLOCKS 5 database represents the initial database used for the publication of the BLOSUM [12] and RBLOSUM matrices [19]. The BLOCKS 13+ covers a larger sequence space and was reported to produce better performing matrices than those created with BLOCKS 5 [6]. BLOCKS 14.3 represents the latest BLOCKS release as of April 2007. This release spans over the largest sequence space available in BLOCKS and represents a more conserved starting point for the parametrization of evolutionary models, such as substitution matrices. We added the labels 5.0, 13+ and 14.3 as subscripts to the matrix names to distinguish from which BLOCKS version a matrix is derived.

For the calculation of the original BLOSUM variants, we chose clustering thresholds of 50 and 62, since the BLOSUM50 _5.0_ and especially BLOSUM62 _5.0_ are two of the most commonly used BLOSUM matrices. For example, these are used as default matrices in SSEARCH [22] and BLAST [21]. Since two substitution matrices can only be properly compared if the difference of their relative entropies is small [24], we adapted the clustering values of the RBLOSUM and CorBLOSUM variants according to the BLOSUM50 and BLOSUM62 variants created from the three different BLOCKS versions. These 18 matrices assessed in our study, their clustering values, relative entropies and matrix scales based on unrounded log-odd scores are listed in Table 1.

**Table 1:** Overview of the matrices assessed in this study and their respective clustering values, relative entropies and corresponding scale in bits per unit

Matrix	Clust. value	Rel. entropy	Bit units
BLOSUM50 _5.0_	50	0.4808	1/3
RBLOSUM52 _5.0_	52	0.4918	1/3
CorBLOSUM49 _5.0_	49	0.4849	1/3
BLOSUM62 _5.0_	62	0.6979	1/2
RBLOSUM64 _5.0_	64	0.7003	1/2
CorBLOSUM61 _5.0_	61	0.6939	1/2
BLOSUM50 _13+_	50	0.2430	1/4
RBLOSUM59 _13+_	59	0.2410	1/4
CorBLOSUM57 _13+_	57	0.2479	1/4
BLOSUM62 _13+_	62	0.3672	1/3
RBLOSUM69 _13+_	69	0.3601	1/3
CorBLOSUM66 _13+_	66	0.3653	1/3
BLOSUM50 _14.3_	50	0.1509	1/5
RBLOSUM59 _14.3_	59	0.1477	1/5
CorBLOSUM57 _14.3_	57	0.1515	1/5
BLOSUM62 _14.3_	62	0.2685	1/4
RBLOSUM69 _14.3_	69	0.2662	1/4
CorBLOSUM67 _14.3_	67	0.2636	1/4

Notably, the difference in the clustering thresholds is rather small for matrices based on BLOCKS 5 when compared to those based on BLOCKS 13+ and BLOCKS 14.3. This effect is induced by the different sequence compositions in the different BLOCKS releases. While the BLOCKS 5 release only provides 27,102 sequences for the matrix calculation, the BLOCKS 13+ provides 663,288 sequences and the even larger BLOCKS 14.3 database 6,739,916 sequences. Similarly, the composition of the database influences the relative matrix entropy. Whereas the entropy of the matrices which originate from BLOCKS 5 database is rather high, the distribution of substitution events (i.e. the joint distribution) in the BLOCKS 13+ and BLOCKS 14.3 are closer to an independent event (i.e. the product of the marginals) and hence the relative substitution matrix entropy is smaller.

### Databases

Analogous to previous studies [6, 19, 20], we chose the ASTRAL database as basis for our performance analysis. The ASTRAL database [14, 15] serves as a gold standard for the assessment of homology search performance and parameter selection [6, 13, 18, 19]. The database itself is a subset of the SCOP/SCOPe databases [16, 17] and consists of structural alignments [14, 15] based on the hand-curated SCOP classification.

As mentioned earlier, the performance study by Styczynski et al. [19] was solely based on the ASTRAL40 1.69 release with less than 40 % identical sequences. In addition, we tested all generated substitution matrices against all available ASTRAL database releases (versions 1.55 to 2.06). Inspired by Angermüller et al. [25], we used for each release three different sequence similarity thresholds (20, 40 and 70 *%*) resulting in 51 separate benchmarks. In the following, we use the terms ASTRAL20, ASTRAL40 and ASTRAL70 to distinguish between these three similarity based subsets. Additionally, we use the terms SCOP or SCOPe based ASTRAL datasets to refer to ASTRAL versions 1.55 to 1.75 and 2.01 to 2.06, respectively. Here, we would like to note, that SCOP based ASTRAL releases are entirely manually curated while SCOPe releases are based on a semi-automated approach for the database generation.

This wide variety of databases allows for the assessment of the effect of improving sequence space coverage and different database compositions on matrix performance.

### Search methods

In order to evaluate the performance of the different substitution matrices on the different ASTRAL databases, we conducted a homology search for each of the 51 ASTRAL databases against itself. Here, we used the Smith-Waterman alignment algorithm implemented in SSEARCH (version 36.3.6d) [22], as SSEARCH has been shown to possess higher accuracy than BLAST in assessing the performance of different substitution matrices [12, 18, 19].

To address the potential bias from suboptimal gap penalty settings on the matrix performance, we varied the gap open penalty between 5 and 20 in spacings of 1 and the gap extension penalty between 1 and 2. These penalties correspond to commonly used parameter settings in homology search tools (BLAST [21] and SSEARCH [22]) and previous performance studies such as [6]. For each combination of matrix, gap open and gap extension penalty, we obtained a list of homologs found for each sequence in the benchmarked ASTRAL release ordered by their respective *E*-value. The best performing gap parameter set for each matrix on each of the tested ASTRAL databases are listed in Additional file 2.

### Performance evaluation

In analogy to previous assessments [6, 19], we used the coverage measure $\mathcal {Q}$ to evaluate the performance of the different matrix/gap combinations. $\mathcal {Q}$ represents the fraction of true positives found in the search results after applying an *E*-value thresholding based on the errors per query (epq) measure from [6, 18]. A widely used toolkit [6, 19, 20], to calculate the coverage measure from SSEARCH results is the PSCE toolkit by Green et al. [18]. In order to handle the large amount of SSEARCH results generated in our study, we used our own CoverageCalculator tool, a performance optimized reimplementation of the PSCE toolkit. The source code of this tool is available at [26].

In our CoverageCalculator, a search result is considered as a true positive relation, if the superfamily annotations, as provided by the ASTRAL database, are identical for the query and the reported sequence. In order to mitigate potential bias from different superfamily sizes, we used the quadratic normalized coverage $\mathcal {Q}_{\text {quad}}$ (Eq. 1) as the average of true positive relations found per superfamily [6]:

(1) $$ \mathcal{Q}_{\text{quad}} = \frac{1}{S}\sum\limits_{i=1}^{S} \frac{t_{\mathrm{i}}}{({s_{i}^{2}} - s_{i})}   $$

Here, *t*_*i*_ is the number of true positive relations found for a superfamily *i* with *s*_*i*_ sequences. *S* is the number of superfamilies in the database.

The *E*-value threshold for the filtering is selected adaptively, depending on the average number of false positive relations remaining in all search results after applying the threshold. A search result is considered a false positive relation, if its superfamily annotation does not match the annotation of the query sequence. This is contrary to the PSCE toolkit, where search results with different superfamily but same fold annotation are ignored in the coverage calculation since their evolutionary relationship is unknown. Hence, our CoverageCalculator takes all reported results into account and thus is not overestimating the “real” coverage by skipping unknown but real false positive relations within the same fold. Since the true evolutionary relationship between the superfamilies is not known, this may underestimate the “real” coverage, but consistently assumes that all superfamilies are not related. Hence, the coverages reported here, represent the lower bound for substitution matrix performance.

In our study, we set the maximum number of errors to 0.01 epq in accordance with previous studies [6, 13, 18, 19]. This corresponds to a maximum of one false positive relation identified per 100 queries on average for the entire database. For example, the search results of ASTRAL40 1.69 database with its 7290 sequences are filtered to contain no more than 72 false positives in total.

In order to evaluate the statistical significance of the performance results for the tested matrix/gap combinations, we used Concerted Bayesian bootstrapping [6, 18], where sequence weights are derived from a Dirichlet distribution. This method effectively analyzes the influence of slight changes in the database composition on the resulting coverage values. Applying the quadratic coverage normalization to the Concerted Bayesian bootstrapping yields the following equations for one bootstrap:

(2a) $$\begin{array}{*{20}l} \widehat{\mathcal{Q}}_{i} &= \sum\limits_{j=1}^{s_{i}}\sum\limits_{m=1}^{N_{j}} \delta(\theta_{j},\theta_{m}) w_{j} w_{m}  \end{array} $$

(2b) $$\begin{array}{*{20}l} \mathcal{W}_{i} &= \sum\limits_{k=1}^{s_{i}}\sum\limits_{l=1}^{s_{i}} w_{k} w_{l} - \sum\limits_{k=1}^{s_{i}} (w_{k})^{2}  \end{array} $$

(2c) $$\begin{array}{*{20}l} \widehat{\mathcal{Q}}_{\text{quad}} &=\frac{1}{S}\sum\limits_{i=1}^{S} \frac{\widehat{\mathcal{Q}}_{i}}{\mathcal{W}_{i}}  \end{array} $$

In Eq. 2a, *w*_*j*_ represents the weight of the *j*th query sequence of superfamily *i*. *θ*_*j*_ represents its superfamily annotation. Likewise, *θ*_*m*_ denotes the superfamily of the *m*th query results for the *j*th sequence with the weight *w*_*m*_. *δ*(*θ*_*j*_,*θ*_*m*_) is the Kronecker delta, returning 1 if *θ*_*j*_ and *θ*_*m*_ are equal, i.e. if both sequences are members of the same superfamily, and zero otherwise. *N*_*j*_ is the number of homologs found for the query sequence and *s*_*i*_ denotes the sequence count of the *i*th superfamily.

Thus, Eq. 2a describes the unnormalized coverage for the *i*th superfamily — all *found* “true positive” relations. Equation 2b is the quadratic normalization for the *i*th superfamily, i.e. all *possible* positive interactions for the *i*th superfamily. Summing over all relative coverages for the *S*-numbered superfamilies (Eq. 2c) returns the quadratic normalized coverage for a single bootstrap.

The significance of the coverage difference of two matrix/gap combinations is tested by calculating a *Z*-score from a two-sample parametric means test using the variance from the two corresponding bootstrap distributions [18]. Hereby, the *Z*-score measures the significance of the difference of the two underlying distributions (see Eq. 3).

(3) $$ Z_{p,q} = \frac{\mathcal{\bar{Q}}_{p} - \mathcal{\bar{Q}}_{q}}{\sqrt{\frac{{\sigma^{2}_{p}} + {\sigma^{2}_{q}}}{N}}}   $$

For two different matrix/gap combinations *p* and *q*, $\mathcal {\bar {Q}}_{p}$ and $\mathcal {\bar {Q}}_{q}$ represent the mean of the bootstrap coverages calculated for the *p*th and *q*th matrix/gap combinations at an errors per query (epq) of 0.01. ${\sigma ^{2}_{p}}$ and ${\sigma ^{2}_{q}}$ correspond to the variance of the underlying bootstrap coverage distributions. *N* represents the number of bootstrap rounds.

In our study, we set the number of bootstrap rounds for each matrix/gap combination to 500 as previously suggested [6]. We consider differences with *Z*≥1.96 as significant which corresponds to the 97.5 percentile.

## Results and discussion

### Matrix differences

**Fig. 1:**
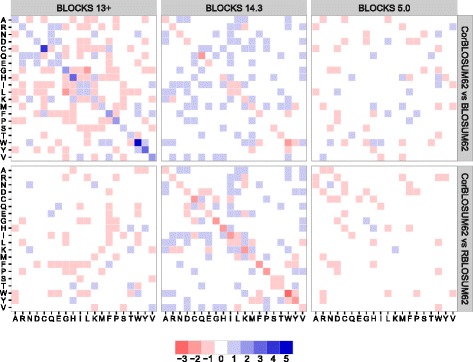
Comparison of matrix entries using the same clustering value 62. Shown are the differences of BLOSUM62 and RBLOSUM62 to CorBLOSUM62 for BLOCKS 5, BLOCKS 13+ and BLOCKS 14.3. Blue tiles represent matrix entries where the respective CorBLOSUM62 values are larger than entries of the compared matrix. Red tiles represent the opposite. While differences for BLOCKS 5 based substitution matrices only range from −1 to 1, the range of these differences is substantially larger for newer BLOCKS versions

In order to assess the impact of the code corrections (see Additional file 1 and [19]) we derived all three matrix variants from the here tested BLOCKS versions using the same clustering value. Exemplary, Fig. 1 highlights the difference in the respective matrix entries for a clustering value of 62. Here, we can clearly see numerous changes between the matrices created by the three algorithms. While differences for BLOCKS 5 based substitution matrices are in the range of −1 to 1, BLOCKS 13+ and BLOCKS 14.3 based matrices can differ to a much greater extend (ranging from −3 to 5) eventually implying a 10^5^ fold change in frequency counts. Thus, changes in the matrices cannot exclusively be related to rounding issues, indicating substantially different algorithms.

**Fig. 2:**
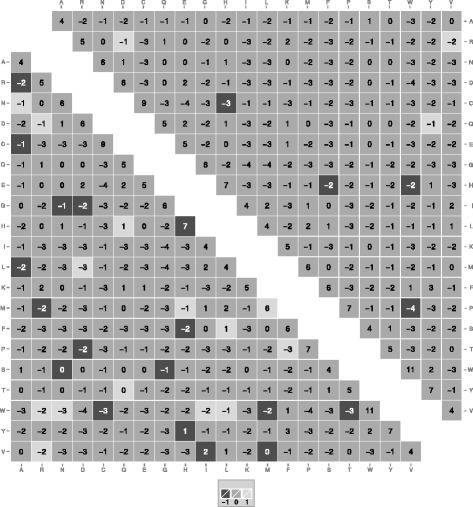
Comparison of CorBLOSUM61 _5.0_ with BLOSUM62 _5.0_ and RBLOSUM64 _5.0_. Differences between CorBLOSUM61 _5.0_ and BLOSUM62 _5.0_ are displayed in the lower triangle and those between CorBLOSUM61 _5.0_ and RBLOSUM64 _5.0_ in the upper triangle, with CorBLOSUM61 _5.0_ values shown. Light gray tiles represent entries where the CorBLOSUM61 _5.0_ matrix is one log-odd score point higher than the compared matrix, whereas dark gray represent a one point lower score of CorBLOSUM61 _5.0_ matrix. Noticeably, the CorBLOSUM correction introduces further changes into the RBLOSUM64 _5.0_ matrix (*upper triangle*) which results in numerous value adjustments when compared to the BLOSUM62 _5.0_ matrix (*lower triangle*)

To properly assess the performance difference between the three different substitution matrix types it is necessary to observe their capabilities at a similar relative entropy level [24]. Here, we compared for example the BLOCKS 5 based matrices CorBLOSUM61 _5.0_, BLOSUM62 _5.0_ and RBLOSUM64 _5.0_ (see Fig. 2). On one hand, a total of 31 matrix entries are different between the CorBLOSUM61 _5.0_ and BLOSUM62 _5.0_ (i.e. 14.8 *%*), with 17 entries being reduced (see Fig. 2, lower triangle). On the other hand, only 7 entries differ between CorBLOSUM61 _5.0_ and RBLOSUM64 _5.0_, with three entries being larger in absolute value. The comparison of the matrices comparable to BLOSUM50 _5.0_ is shown in Additional file 3: Figure S1. The smaller number of differences between RBLOSUM64 _5.0_ and CorBLOSUM61 _5.0_ are not unexpected, as the RBLOSUM correction is also included in the CorBLOSUM algorithm. However, the number of differences between CorBLOSUM and RBLOSUM type matrices increases for other BLOCKS versions. The large differences between CorBLOSUM- and BLOSUM-type matrices observed for BLOCKS 5 can also be observed for the other two BLOCKS releases. The complete overview of differences for all entropy levels assessed in this paper is given in Additional file 4: Figure S2 and Additional file 5: Figure S3. The comparison of matrices based on a similar entropy level further highlights that the three algorithms create substantially different substitution matrices.

### General matrix performance overview

**Fig. 3:**
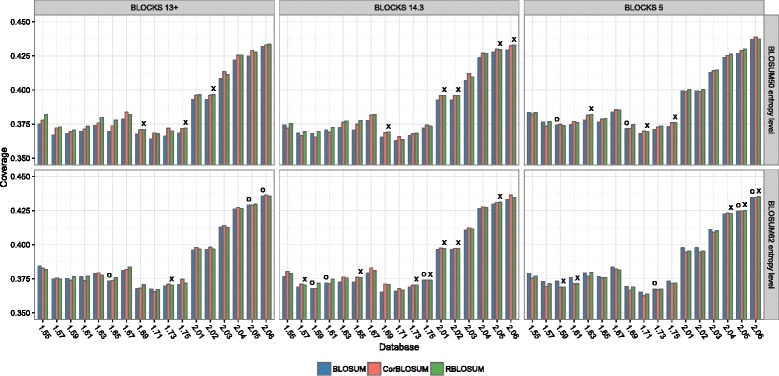
Progression of the maximum achieved coverage of CorBLOSUM-, RBLOSUM- and BLOSUM-type matrices for all ASTRAL40 test databases. The upper row shows the results for the respective BLOSUM50 entropy level, the lower row for BLOSUM62 entropy level. Insignificant coverage differences between CorBLOSUM and BLOSUM are indicated by an **O** and between CorBLOSUM and RBLOSUM by a small an **X** above the bars. The corresponding gap parameter settings are listed in Additional file 2. Notably, the coverage increases for all tested substitution matrices dramatically with the introduction of the semi-automatic database generation of SCOPe. For the BLOSUM50 entropy level, CorBLOSUM-type matrices performed at least as good as their BLOSUM counterparts in ∼84 *%* of all tested scenarios and in ∼49 *%* showed a similar or better performance than the RBLOSUM-type matrices. For the BLOSUM62 entropy level CorBLOSUM matrices showed equally as good or better performance than BLOSUM in ∼67 *%* while improving performance over RBLOSUM in ∼60 *%* of all analyzed ASTRAL40 scenarios

Using the above described benchmarking method we compared the different matrix variants BLOSUM, RBLOSUM and CorBLOSUM. The achieved coverage values for these matrices and their respective best gap parameter settings are shown in Fig. 3, Additional file 6: Figure S4 and Additional file 7: Figure S5. For all test scenarios we consider performance differences with Z-scores <1.96 as insignificant and thus assume matrix performance to be almost equal. In cases where the coverage difference between a BLOSUM- and CorBLOSUM-type matrix is insignificant as denoted by its corresponding *Z*-score value, an **O** is displayed above the bar. For the CorBLOSUM/RBLOSUM comparison, we highlight this with a small **X**. The underlying *Z*-scores for estimating the significance of these coverage differences are shown for completeness in Additional file 8: Figure S6, Additional file 9: Figure S7 and Additional file 10: Figure S8.

In order to obtain a general overview, we counted the number of times a specific CorBLOSUM matrix performed equally or better than its corresponding BLOSUM counterpart. Considering all test scenarios, substitution matrices computed with the CorBLOSUM algorithm performed at least as good as their BLOSUM counterparts in ∼75 *%* of the time. On SCOPe based ASTRAL releases this percentage increased to ∼86 *%*.

Since we cannot directly compare the performance of substitution matrices derived from different BLOCKS versions due to their relative entropies, we compared the performance of each substitution matrix on all three similarity based ASTRAL subsets in identical manner to the above described. Cases where CorBLOSUM matrices performed at least as good as their corresponding BLOSUM variants derived from the three different BLOCKS versions are shown in percent in Table 2. Here, the CorBLOSUM matrices performed better than the BLOSUM matrices with one interesting exception, the original BLOSUM62 _5.0_ matrix. This matrix still performed better than its CorBLOSUM61 _5.0_ counterpart in most of the cases on the ASTRAL20 and ASTRAL40 subsets.

**Table 2:** Comparison of CorBLOSUM- with BLOSUM-type matrices

	ASTRAL subset	BLOSUM50	BLOSUM62
		entropy level	entropy level
BLOCKS 13+	ASTRAL20	94.12 %	58.82 %
	ASTRAL40	100 %	76.47 %
	ASTRAL70	100 %	82.35 %
BLOCKS 14.3	ASTRAL20	76.47 %	76.47 %
	ASTRAL40	76.47 %	100 %
	ASTRAL70	88.24 %	70.59 %
BLOCKS 5	ASTRAL20	70.59 %	23.53 %
	ASTRAL40	76.47 %	23.53 %
	ASTRAL70	100 %	58.82 %

Shown in percent is the relative frequency for which a CorBLOSUM matrix performed at least as good as its BLOSUM counterpart

Although, the achieved coverage range differs widely between the ASTRAL20, ASTRAL40 and ASTRAL70 subsets, our results show a specific performance pattern within each identity subset regardless of the BLOCKS version and entropy level used for the computation of the matrices. For ASTRAL40 and ASTRAL70, the coverage increases drastically for ASTRAL versions based on SCOP (version ≤1.75) to those based on SCOPe (version ≥2.01). Interestingly, this trend cannot be observed for ASTRAL20. In the following sections, we discuss the matrix performances on each of the three different similarity based ASTRAL subsets in detail.

### Matrix performance on ASTRAL40

The resulting coverage values for all tested ASTRAL40 versions and substitution matrices are shown in Fig. 3. The reported values reflect the respective best matrix / gap parameter combinations. The *Z*-scores representing the statistical significance of the coverage differences are shown in Additional file 8: Figure S6.

For the ASTRAL40 subset, a general performance trend can be observed for all assessed relative entropy levels. Starting from ASTRAL release 1.57 the performance increases steadily until ASTRAL 1.69, the database used by Styczynski et al. to measure the RBLOSUM performance. Here, a drastic drop in the coverages can be observed. From ASTRAL 1.71 the coverages continue to steadily increase with a very large increment upon the introduction of SCOPe at ASTRAL 2.01. The highest coverage over all entropy levels, BLOCKS versions and ASTRAL releases was obtained for CorBLOSUM49 _5.0_ on ASTRAL 2.06 with a coverage of 0.4389 at a gap open/extension penalty of 15/1.

For BLOCKS 5 derived substitution matrices at a matrix entropy level of ∼0.7 bit, the original, inaccurate BLOSUM62 _5.0_ dominates the corrected variants for nearly every ASTRAL release but the newest three. For these, CorBLOSUM61 _5.0_ and RBLOSUM64 _5.0_ performed at least as well as BLOSUM62 _5.0_ at a statistical significant level. Our results for the ASTRAL 1.69 database are in concordance with the results published in the RBLOSUM study [19] — i.e. the BLOSUM62 _5.0_ significantly outperforms the RBLOSUM64 _5.0_. Interestingly, the used BLOCKS version significantly influences this performance difference as RBLOSUM matrices derived from BLOCKS 13+ and BLOCKS 14.3 outperform their BLOSUM counterparts.

The CorBLOSUM49 _5.0_ showed higher coverages than the BLOSUM50 _5.0_ for all databases but the oldest ASTRAL and the oldest SCOPe derived ASTRAL databases 2.01 and 2.02. In general, BLOSUM50 _5.0_ entropy level matrices achieve higher coverages than those at the BLOSUM62 _5.0_ entropy level. This cannot be observed for BLOCKS 13+ and BLOCKS 14.3.

For these, the CorBLOSUM57 _13+_ and CorBLOSUM67 _14.3_ consistently outperformed their BLOSUM counterparts on all test databases. CorBLOSUM66 _13+_ and CorBLOSUM57 _14.3_ achieved a coverage at least as high as the BLOSUM in ∼76 *%* of the tested scenarios. For all SCOPe derived ASTRAL datasets CorBLOSUM substitution matrices outperformed their respective BLOSUM variant.

The comparison between CorBLOSUM- and RBLOSUM-type matrices showed overall mixed results. Notably, CorBLOSUM matrices derived from BLOCKS 13+ and BLOCKS 14.3 achieved higher coverages than RBLOSUM matrices in ∼83 *%* of the analyzed SCOPe based datasets.

### Matrix performance on ASTRAL20 and ASTRAL70

Overall, the matrix performances on the ASTRAL70 subset showed the highest coverages followed by ASTRAL40 and ASTRAL20. On the latter the reported coverage values are well below 0.17 which indicates that all tested substitution matrices do not perform well on diverse sequence datasets.

While the coverage trend in respect to the ASTRAL releases on the ASTRAL70 subset is similar to ASTRAL40, a very different trend can be observed for the ASTRAL20 subset (see Additional file 6: Figure S4 and Additional file 7: Figure S5). On SCOPe based ASTRAL70 releases CorBLOSUM matrices outperformed their BLOSUM counterparts in ∼92 *%* of the test datasets, over all BLOCKS versions and entropy levels. Similarly, on the ASTRAL20 subset CorBLOSUM variants achieved a rating of ∼94 *%* in comparison to BLOSUM at the BLOSUM50 entropy level. On the BLOSUM62 entropy level CorBLOSUM outperformed BLOSUM in ∼66 *%* of the times. A detailed discussion of the matrix performance on the ASTRAL20 and ASTRAL70 subsets is given in Additional file 1.

## Conclusion

In this paper, we presented an additional error correction to the BLOSUM code resulting in a new and significantly different matrix computation algorithm. The matrices created by our CorBLOSUM algorithm are substantially different from (R)BLOSUM matrices and outperformed the original BLOSUM matrices in ∼75 *%* of all 51 test scenarios. On up-to-date SCOPe based ASTRAL releases, the current gold standard for homology search performance assessment, the CorBLOSUM matrices outperformed their BLOSUM counterparts in ∼86 *%* of the cases. On these databases, the CorBLOSUM matrices also achieved the highest reported coverages for all three ASTRAL similarity subsets when compared with their BLOSUM counterparts.

The aim of this study was not to assess optimal parameters for homologous sequence search, such as the best matrix/gap-parameter combination. Nevertheless, this is an interesting question which should be addressed in the future, especially since our study showed that the relative entropy of substitution matrices is not necessarily an indicator for matrix performance.

Our results for the BLOSUM62 _5.0_ vs. RBLOSUM64 _5.0_ setup concur with previous findings [19]. There, the test covered only a very specific scenario (ASTRAL40 1.69) in which the RBLOSUM64 _5.0_ was outperformed by the BLOSUM62 _5.0_. These previous results would have been quite different if at that time other available BLOCKS and ASTRAL databases had been used. RBLOSUM matrices tested in this study performed in most of the times equally or better than their BLOSUM counterparts. Our study showed that for the RBLOSUM/CorBLOSUM comparison no consistent trend can be observed for older ASTRAL releases prior to 2.01, as RBLOSUM and CorBLOSUM matrices each being superior in ∼50 *%* of the cases. However, on databases with increased sequence and structure space coverage — as provided by SCOPe based ASTRAL versions — CorBLOSUM-type matrices achieved higher coverages than the RBLOSUM matrices in ∼74 *%* of the tests.

Furthermore, our study revealed two contradicting effects: on the one hand, matrices with very similar entropies show a statistically significant differing performance. On the other hand, we also showed that matrices with very different entropies and matrix scales can achieve similar coverages. The latter effect is apparently enhanced by increasing sequence similarity within superfamilies and the database itself. This raises an interesting question for further research on the influence of changes in database composition on its respective searchability. We conclude that the CorBLOSUM algorithm introduced here fixes errors of the original BLOSUM implementation and that the resulting matrices perform better for homologous sequence search. Hence, we encourage the usage of CorBLOSUM matrices for this specific task.

## Availability of data and material

The ASTRAL databases supporting the conclusions of this article are available at http://scop.berkeley.edu/astral/. The reported coverage values supporting the conclusions of this article are included within the article and its additional files. The matrices discussed in this article are available at http://www.cbs.tu-darmstadt.de/CorBLOSUM.

Information about the CoverageCalculator tool:**Project name:**CoverageCalculator**Project home page:**http://www.cbs.tu-darmstadt.de/CorBLOSUM**Operating system(s):** Linux (Tested on Ubuntu 14.04)**Programming language:** C++11**Other requirements:** OpenMP 3.0**License:** GNU GPLv3

## Additional files

Additional file 1 Detailed description of the CorBLOSUM error correction with analysis of its impact related to BLOCKS 5 and BLOCKS 14.3 database composition. Furthermore, a detailed discussion of the matrix performances on ASTRAL70 and ASTRAL20 subsets is given. (PDF 189 kb)

Additional file 2 List of best performing matrix/gap parameter combinations. (CSV 47.6 kb)

Additional file 3
**Figure S1.** Plot of the differences in entries for BLOSUM50 _5.0_, RBLOSUM52 _5.0_ and CorBLOSUM49 _5.0_ with similar entropy. Differences of CorBLOSUM49 _5.0_ and BLOSUM50 _5.0_ displayed in the lower triangle and of CorBLOSUM49 _5.0_ and RBLOSUM52 _5.0_ in the upper triangle, with CorBLOSUM49 _5.0_ values shown. Light gray tiles represent entries where the CorBLOSUM49 _5.0_ matrix is one log-odd score point higher than the compared matrix, whereas dark gray represent a 1 point lower score of CorBLOSUM49 _5.0_ matrix. White squares represent entries where the CorBLOSUM49 _5.0_ is two points higher than the compared matrix. Noticeably, the CorBLOSUM correction introduces further changes into the RBLOSUM52 _5.0_ matrix (upper triangle) which results into numerous value adjustments when compared to the BLOSUM50 _5.0_ matrix (lower triangle). (PDF 18.3 kb)

Additional file 4
**Figure S2.** Comparison of all analyzed CorBLOSUM matrices with their corresponding counterparts for all three BLOCKS databases at their respective BLOSUM62 entropy level. Entries for which the CorBLOSUM is higher than the compared matrix are displayed in blue and red vice versa. White entries symbolize no change in value. Noticeably, the CorBLOSUM-type matrices differ to a great extend from the BLOSUM-type matrices, while the changes between CorBLOSUM and RBLOSUM are fewer but still numerous. (PDF 16.3 kb)

Additional file 5
**Figure S3.** Comparison of all analyzed CorBLOSUM matrices with their corresponding counterparts for all three BLOCKS databases at their respective BLOSUM50 entropy level. Entries for which the CorBLOSUM is higher than the compared matrix are displayed in blue and red vice versa. White entries symbolize no change in value. Compared to Additional file 4: Figure S2 the differences of the CorBLOSUM-type matrices to BLOSUM-type matrices increase in number and extend, while the frequency of changes compared to RBLOSUM is similar. (PDF 16.6 kb)

Additional file 6
**Figure S4.** Progression of the maximum achieved coverage of CorBLOSUM-, RBLOSUM- and BLOSUM-type matrices for all ASTRAL70 test databases. The upper row shows the results for the respective BLOSUM50 entropy level, the lower row for BLOSUM62 entropy level. An insignificant coverage difference between CorBLOSUM and BLOSUM is indicated by an **O** and between CorBLOSUM and RBLOSUM by an **X**. The corresponding gap parameter settings are listed in Additional file 2. Similar to the ASTRAL40 test scenarios, a drastic increase in coverage can be observed for SCOPe based ASTRAL databases. For the BLOSUM50 entropy level, CorBLOSUM-type matrices performed at least as good as their BLOSUM counterparts in ∼94 *%* of all tested scenarios and in ∼51 *%* showed a similar or better performance than the RBLOSUM-type matrices. For the BLOSUM62 entropy level CorBLOSUM matrices showed equally as good or better performance than BLOSUM in ∼75 *%* while improving performance over RBLOSUM in ∼59 *%* of all analyzed ASTRAL70 scenarios. (PDF 9.69 kb)

Additional file 7
**Figure S5.** Progression of the maximum achieved coverage of CorBLOSUM-, RBLOSUM- and BLOSUM-type matrices for all ASTRAL20 test databases. The upper row shows the results for the respective BLOSUM50 entropy level, the lower row for BLOSUM62 entropy level. An insignificant coverage difference between CorBLOSUM and BLOSUM is indicated by an **O** and between CorBLOSUM and RBLOSUM by an **X**. The corresponding gap parameter settings are listed in Additional file 2. The BLOSUM62 entropy level substitution matrices derived from BLOCKS _13+_ and BLOCKS _14.3_ consistently achieved higher coverages than those on the BLOSUM50 entropy level. For the BLOSUM50 entropy level, CorBLOSUM-type matrices performed at least as good as their BLOSUM counterparts in ∼80 *%* of all tested scenarios and in ∼53 *%* showed a similar or better performance than the RBLOSUM-type matrices. For the BLOSUM62 entropy level CorBLOSUM matrices showed equally as good or better performance than BLOSUM in ∼49 *%* while improving performance over RBLOSUM in ∼70 *%* of all analyzed ASTRAL20 scenarios. (PDF 9.69 kb)

Additional file 8
**Figure S6.** *Z*-scores for the coverage comparison of CorBLOSUM with BLOSUM and RBLOSUM based on Bayesian bootstrap for the ASTRAL40 datasets. (PDF 8.61 kb)

Additional file 9
**Figure S7.** *Z*-scores for the coverage comparison of CorBLOSUM with BLOSUM and RBLOSUM based on Bayesian bootstrap for the ASTRAL70 datasets. (PDF 8.55 kb)

Additional file 10
**Figure S8.** *Z*-scores for the coverage comparison of CorBLOSUM with BLOSUM and RBLOSUM based on Bayesian bootstrap for the ASTRAL20 datasets. (PDF 8.91 kb)
