# Supplementary material for: Addressing inaccuracies in BLOSUM computation improves homology search performance
Source: BMC Bioinformatics. 2016 Apr 27;17:189. doi: 10.1186/s12859-016-1060-3 (PMC4849092; doi:10.1186/s12859-016-1060-3)
Supplement: Additional file 1 — Detailed description of the CorBLOSUM error correction with analysis of its impact related to BLOCKS 5 and BLOCKS 14.3 database composition. Furthermore, a detailed discussion of the matrix performances on ASTRAL70 and ASTRAL20 subsets is given. (PDF 189 kb) [file 12859_2016_1060_MOESM1_ESM.pdf]

Supplemental Material
Addressing inaccuracies in BLOSUM computation improves homology search performance

Martin Hess [1,2,4]***, Frank Keul*** [2,3,4]***, Michael Goesele*** [1] ***& Kay Hamacher*** [2]

[1] *Department of Computer Science, Technische Universität Darmstadt, Graphics, Capture and Massively Parallel Computing, Rundeturmstraße 12, 64283 Darmstadt, Germany.*
[2] *Department of Biology, Technische Universität Darmstadt, Computational Biology and Simulation, Schnittspahnstraße 2, 64287 Darmstadt, Germany.*
[3] *To whom correspondence should be addressed.*
[4] *The authors wish it to be known that, in their opinion, the first two authors should be regarded as joint First Authors.*

Error description

In the BLOSUM matrix computation a specific clustering threshold is computed for each sequence block of the BLOCKS data set via a user specified clustering percentage (defined in `Cluster`) and the block's size (defined in `Block.width`). Through this, a similarity score (`pairs[px].score`) can be calculated for every pair of sequences of the respective block by counting the number of identical residues. This count is then compared to the aforementioned block and clustering percentage specific `threshold` to decide whether two sequences should be — as similar sequences — clustered or not.

```
    // Original threshold calculation
680 int threshold =
    (int)(Cluster*(Block.width))/100;
    ...
    // Clustering decision
728 if (pairs[px].score >= threshold){
      // Cluster sequences
      ...
    }
```

```
    // Corrected threshold calculation
680 float threshold =
    (float)(Cluster*(Block.width))/100f;
    ...
    // Clustering decision
728 if (pairs[px].score >= threshold){
      // Cluster sequences
      ...
    }
```

Supplementary Listing 1: Threshold calculation and clustering decision adapted from lines 680 and 728 of the original `blosum.c` file (available at [1]). **Left)** The original code lines using an integer based clustering threshold. **Right)** The corrected code lines using a floating point based clustering threshold. Differences between both listings are highlighted in red. The loss of precision caused by the integer based threshold can lead to an inaccurate clustering decision in line 728.

Supplementary Listing 1 shows the relevant code parts for the calculation of the clustering threshold and the clustering decision adapted from the original `blosum.c` file. The left side depicts the original code lines using an integer based clustering threshold, which effectively truncates the *real* floating point threshold from line 680 at the integer position by way of an explicit type cast. This loss in precision can lead to an inaccurate clustering decision in the main clustering routine. To avoid these inaccuracies, we use a floating point clustering threshold, as depicted on the right side of Supplementary Listing 1.

In order to quantify the effect of the integer type cast we calculated the relative threshold difference ΔT_{rel} between the correct clustering threshold ($T = \frac{K \cdot l_{\text{block}}}{100}$) and the threshold used in the actual BLOSUM code ($\widehat{T} = \left\lfloor \frac{K \cdot l_{\text{block}}}{100} \right\rfloor$).

For increasing block lengths l_{block} and given integer clustering coefficient K, ΔT_{rel} describes the difference between the clustering thresholds in relation to the block size found in the BLOCKS 5 database [2] with the equation $\Delta T_{\text{rel}} = \frac{T-\widehat{T}}{l_{\text{block}}}$ (see Fig. 1 for $K = 62$). As the BLOCKS 14.3 database consists of two orders of magnitude more sequences than BLOCKS 5 (6739916 sequence entries to BLOCKS 5's 27102), more sequences are incorrectly clustered. Hence, for BLOCKS 14.3 we expect larger differences between the derived BLOSUM and CorBLOSUM matrices.

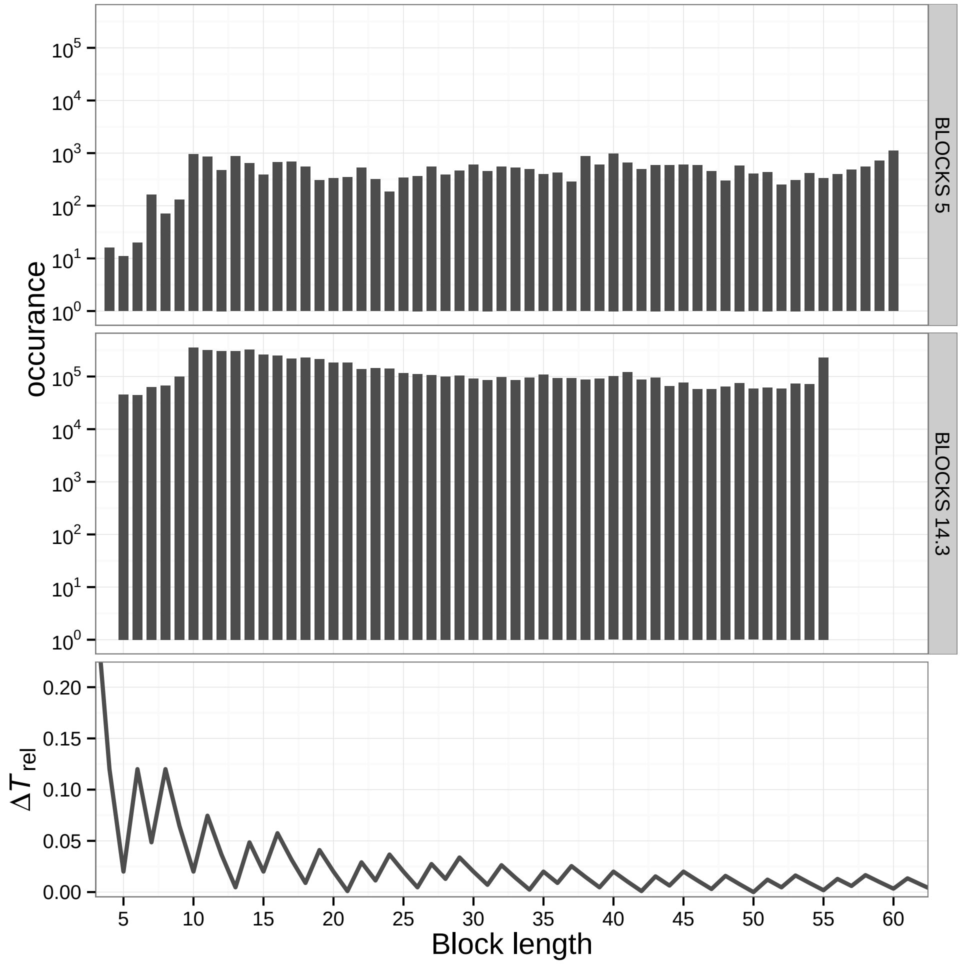

Supplementary Figure 1: Theoretical error indicated by the relative threshold difference ΔT_{rel} for the clustering coefficient $K = 62$. In the lowest panel, the difference ΔT_{rel} between the floating point threshold T and the truncated threshold $\widehat{T}$ is shown for increasing block lengths. The number of sequences found in the BLOCKS 5 and BLOCKS 14.3 databases for these block lengths are depicted in the panels above. For smaller blocks this relative difference is large and vanishes with increasing block length. Note the systematic and therefore biased behavior of ΔT_{rel} as function of the block length.

Matrix Performance on ASTRAL70

The obtained coverage values for the tested ASTRAL70 versions and substitution matrices are shown in Additional figure 4. Similar to the ASTRAL40 results, the reported values represent the respective best matrix / gap parameter combinations with Z-scores shown in Additional figure 7. Again a general performance progression can be identified which is nearly the same as that observed for the ASTRAL40 subset, i.e the performance drop at ASTRAL 1.69 and the large increase starting with the introduction of the SCOPe based ASTRAL 2.01. Here again, the highest coverage was obtained for CorBLOSUM66$_{13+}$ on ASTRAL 2.06 with a coverage of 0.5445 at a gap open/extension penalty of 12/1. In general, the achieved coverages are higher than those on the ASTRAL40 subset. This is expected, since the

average sequence similarity in ASTRAL70 is higher than in ASTRAL40 and all matrices listed here generally favor conservation over substitution. The latter is represented by larger log-odd scores on the matrix diagonal than those on off-diagonals.

For an entropy level comparable to BLOSUM50 matrices, their corresponding CorBLOSUM counterparts performed at least as good in $\sim 96\%$ of all test cases. In particular, the CorBLOSUM coverage for BLOCKS 5 and BLOCKS 13+ was equal or higher in all and for BLOCKS 14.3 in $\sim 88\%$ of the tested databases when compared to their corresponding BLOSUM50 matrix.

In comparison to the three different BLOSUM62 matrices, CorBLOSUM matrices achieved equal or higher coverage values in $\sim 71\%$ of all tested databases. In particular, CorBLOSUM66_{13+} and CorBLOSUM$67_{14.3}$ performed at least as good as the corresponding BLOSUM62 matrices in $\sim 82\%$ and $\sim 71\%$ of all test cases. The BLOCKS 5 derived CorBLOSUM$61_{5.0}$ was still able to achieve a similar or higher rating than BLOSUM$62_{5.0}$ in $\sim 59\%$ of the test databases. Nevertheless, CorBLOSUM$61_{5.0}$ showed here the same performance as the BLOSUM$62_{5.0}$ when considering only SCOPe derived ASTRAL datasets. On these CorBLOSUM matrices outperformed their BLOSUM counterparts in $\sim 92\%$ of the time over all BLOCKS versions and entropy levels. When comparing CorBLOSUM- and RBLOSUM-type matrices no clear statement of performance advantages can be made since both matrix types delivered a similar performance altogether.

Matrix Performance on ASTRAL20

Additional figure 5 shows the coverage values reported for the tested ASTRAL20 versions. The overall coverage progression is different for these databases compared to ASTRAL40 or ASTRAL70 datasets. Nevertheless, an increment in coverage is also observable for SCOPe based ASTRAL versions but starting here at ASTRAL 2.04. The corresponding Z-scores are shown in Additional figure 7. In general, the reported coverage is substantially lower than those observed for ASTRAL40 and ASTRAL70. This indicates that all tested substitution matrices do not perform well on datasets containing only diverse sequences. Here, the highest coverage of only 0.1634 at a gap open and extension penalty of 12/2 was achieved by the RBLOSUM69_{13+} on the oldest ASTRAL release, ASTRAL 1.55. The highest coverage for the newest ASTRAL version was found for the RBLOSUM$52_{5.0}$ with a coverage of 0.1544 at a gap open/extension penalty of 16/1.

At the BLOSUM50 entropy levels the tested CorBLOSUM matrices performed at least as good as their BLOSUM counterparts in $\sim 80\%$ of all scenarios. In detail, the CorBLOSUM coverage for BLOCKS 5 was equal or higher in $\sim 71\%$, in $\sim 94\%$ for BLOCKS 13+ and in $\sim 76\%$ for BLOCKS 14.3 of the tests. On SCOPe derived ASTRAL versions CorBLOSUM variants achieved a rating of $\sim 94\%$ in comparison to BLOSUM.

When compared to the BLOSUM62 variants, CorBLOSUM66_{13+}, CorBLOSUM$67_{14.3}$ and CorBLOSUM$61_{5.0}$ performed at least as good only in $\sim 59\%$, $\sim 76\%$ and $\sim 24\%$ of all test cases. For the latter, BLOSUM$62_{5.0}$ performed best over all but the newest three ASTRAL releases. For these ASTRAL datasets the CorBLOSUM$61_{5.0}$ outperformed the BLOSUM$62_{5.0}$ significantly. When comparing CorBLOSUM- and RBLOSUM-type matrices it is remarkable that for older ASTRAL releases RBLOSUM often performs significantly better than its CorBLOSUM counterpart. Nevertheless, over all BLOCKS versions and entropy levels, CorBLOSUM matrices perform at least as good as RBLOSUM-type matrices in $\sim 63\%$ of all databases. For SCOPe derived ASTRAL sets this percentage increases to $\sim 92\%$.

References

[1] BLOSUM source code. Accessed 18 Sept 2015. ftp://ftp.ncbi.nih.gov/repository/blocks/unix/blosum/blosum.tar.Z

[2] Henikoff, S., Henikoff, J.G.: Automated assembly of protein blocks for database searching. Nucleic Acids Research **19**(23), 6565–6572 (1991)
