# Supplementary figures and images for: Addressing inaccuracies in BLOSUM computation improves homology search performance
Source: BMC Bioinformatics. 2016 Apr 27;17:189. doi: 10.1186/s12859-016-1060-3 (PMC4849092; doi:10.1186/s12859-016-1060-3)

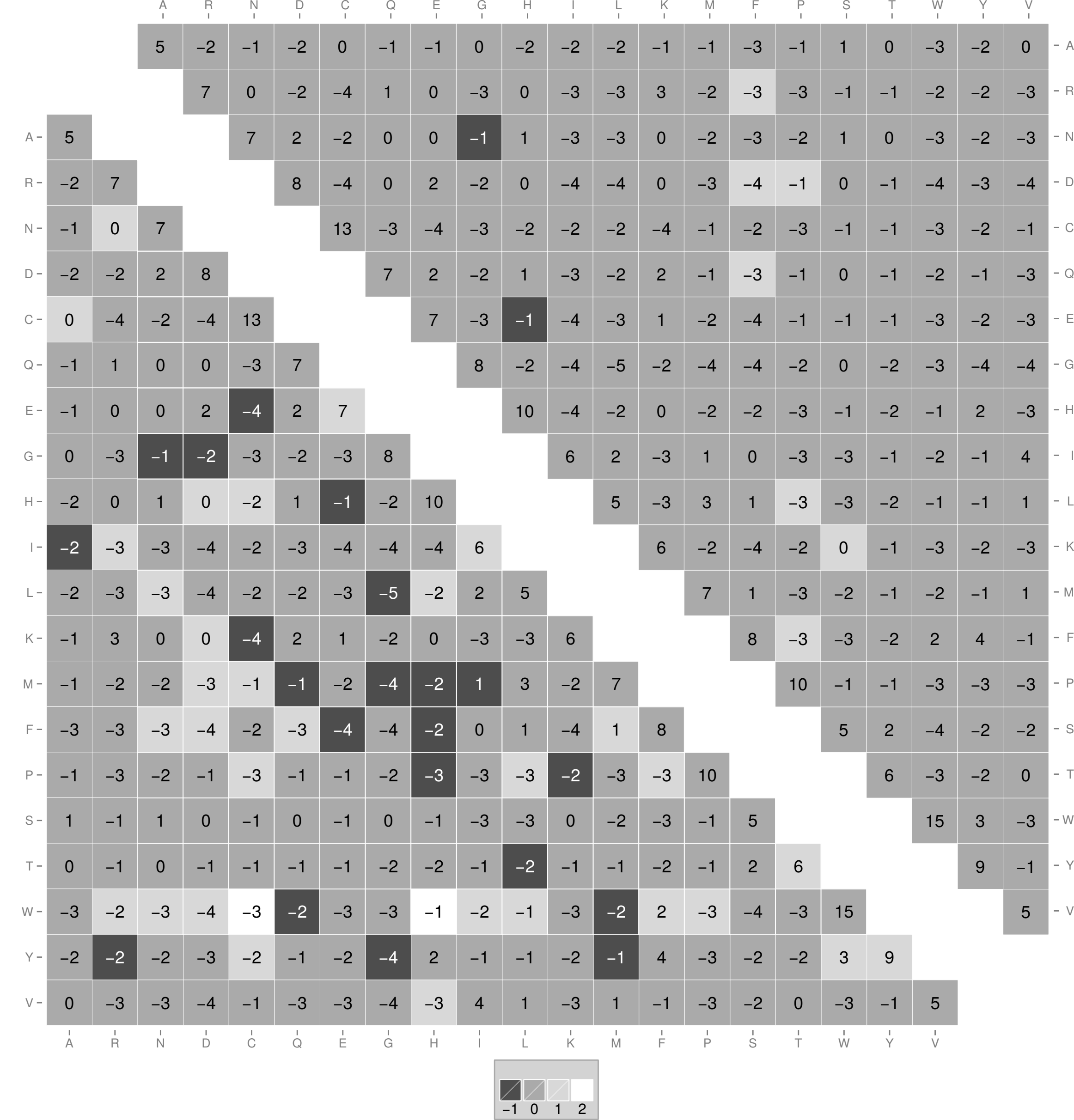

Supplement: Additional file 3 — Figure S1. Plot of the differences in entries for BLOSUM50 5.0, RBLOSUM52 5.0 and CorBLOSUM49 5.0 with similar entropy. Differences of CorBLOSUM49 5.0 and BLOSUM50 5.0 displayed in the lower triangle and of CorBLOSUM49 5.0 and RBLOSUM52 5.0 in the upper triangle, with CorBLOSUM49 5.0 values shown. Light gray tiles represent entries where the CorBLOSUM49 5.0 matrix is one log-odd score point higher than the compared matrix, whereas dark gray represent a 1 point lower score of CorBLOSUM49 5.0 matrix. White squares represent entries where the CorBLOSUM49 5.0 is two points higher than the compared matrix. Noticeably, the CorBLOSUM correction introduces further changes into the RBLOSUM52 5.0 matrix (upper triangle) which results into numerous value adjustments when compared to the BLOSUM50 5.0 matrix (lower triangle). (PDF 18.3 kb) [file 12859_2016_1060_MOESM3_ESM.pdf]

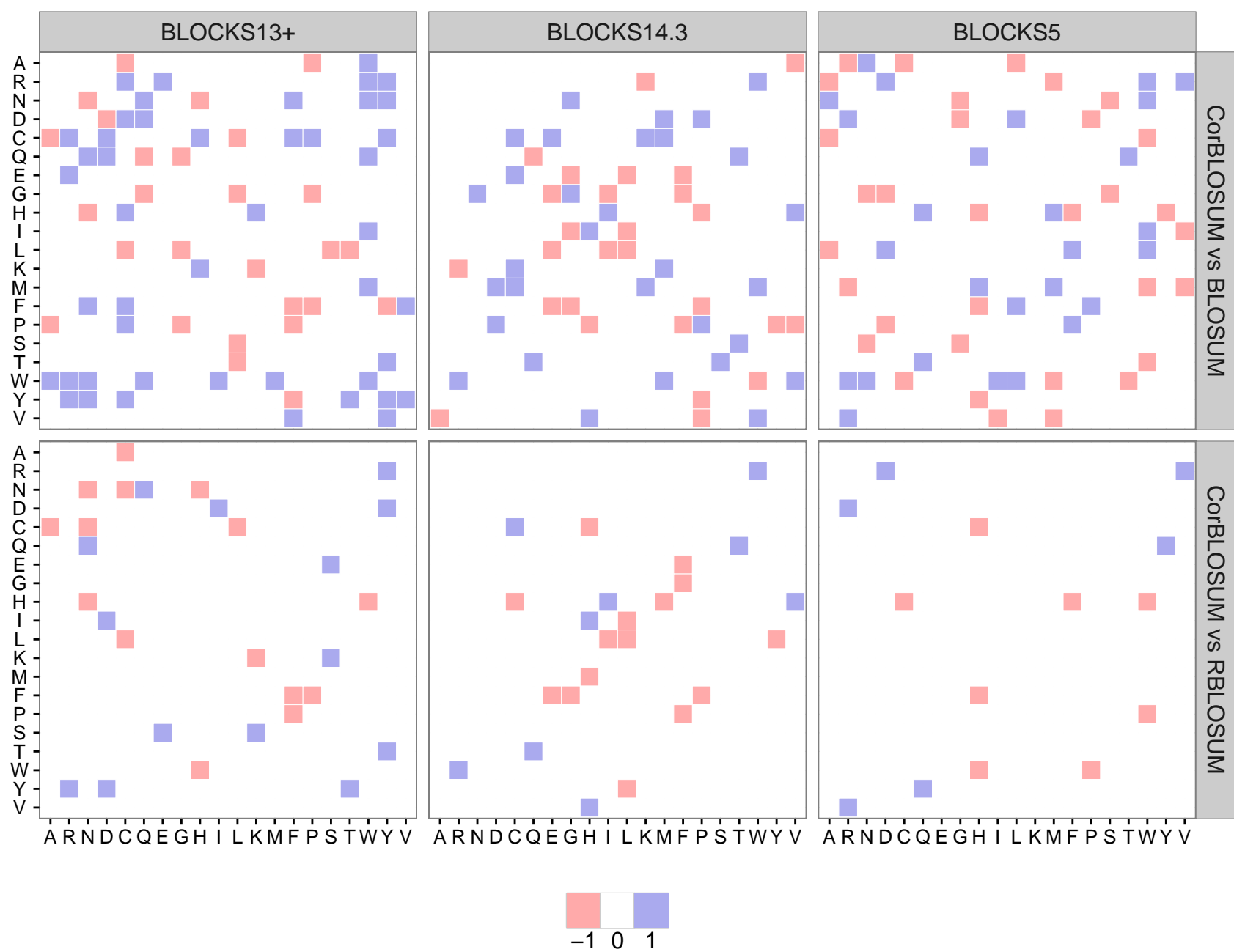

Supplement: Additional file 4 — Figure S2. Comparison of all analyzed CorBLOSUM matrices with their corresponding counterparts for all three BLOCKS databases at their respective BLOSUM62 entropy level. Entries for which the CorBLOSUM is higher than the compared matrix are displayed in blue and red vice versa. White entries symbolize no change in value. Noticeably, the CorBLOSUM-type matrices differ to a great extend from the BLOSUM-type matrices, while the changes between CorBLOSUM and RBLOSUM are fewer but still numerous. (PDF 16.3 kb) [file 12859_2016_1060_MOESM4_ESM.pdf]

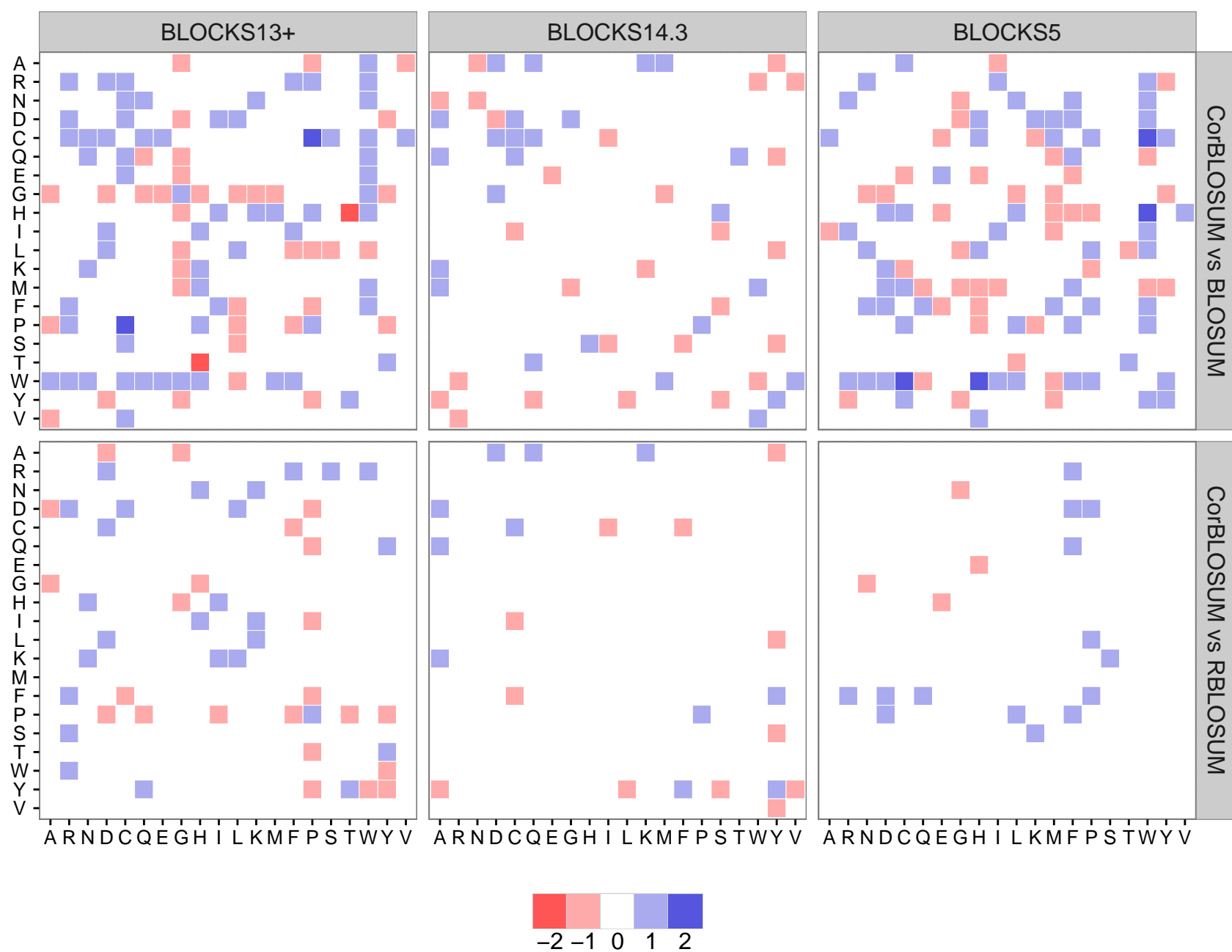

Supplement: Additional file 5 — Figure S3. Comparison of all analyzed CorBLOSUM matrices with their corresponding counterparts for all three BLOCKS databases at their respective BLOSUM50 entropy level. Entries for which the CorBLOSUM is higher than the compared matrix are displayed in blue and red vice versa. White entries symbolize no change in value. Compared to Additional file 4: Figure S2 the differences of the CorBLOSUM-type matrices to BLOSUM-type matrices increase in number and extend, while the frequency of changes compared to RBLOSUM is similar. (PDF 16.6 kb) [file 12859_2016_1060_MOESM5_ESM.pdf]

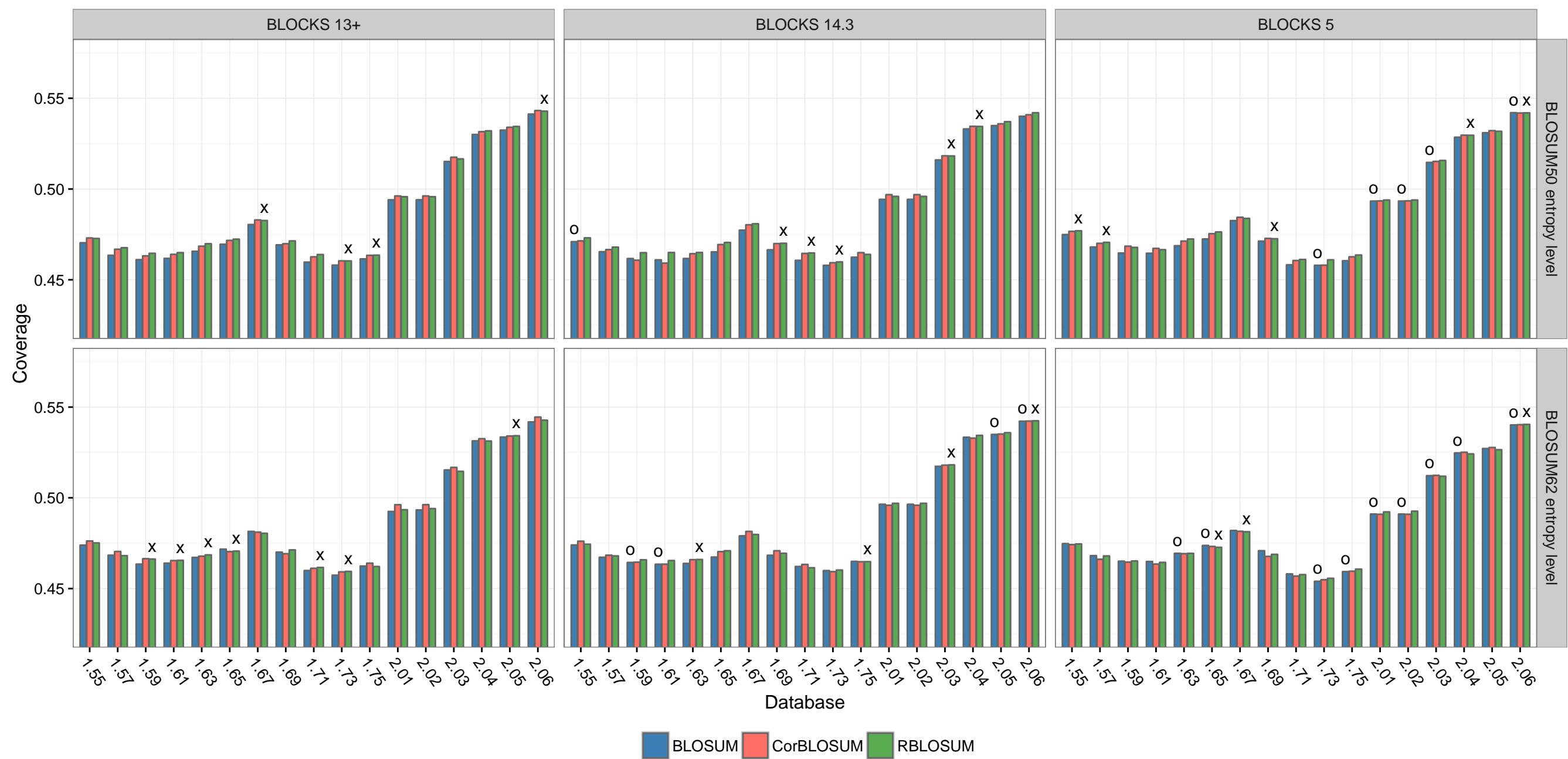

Supplement: Additional file 6 — Figure S4. Progression of the maximum achieved coverage of CorBLOSUM-, RBLOSUM- and BLOSUM-type matrices for all ASTRAL70 test databases. The upper row shows the results for the respective BLOSUM50 entropy level, the lower row for BLOSUM62 entropy level. An insignificant coverage difference between CorBLOSUM and BLOSUM is indicated by an O and between CorBLOSUM and RBLOSUM by an X. The corresponding gap parameter settings are listed in Additional file 2. Similar to the ASTRAL40 test scenarios, a drastic increase in coverage can be observed for SCOPe based ASTRAL databases. For the BLOSUM50 entropy level, CorBLOSUM-type matrices performed at least as good as their BLOSUM counterparts in ∼94 % of all tested scenarios and in ∼51 % showed a similar or better performance than the RBLOSUM-type matrices. For the BLOSUM62 entropy level CorBLOSUM matrices showed equally as good or better performance than BLOSUM in ∼75 % while improving performance over RBLOSUM in ∼59 % of all analyzed ASTRAL70 scenarios. (PDF 9.69 kb) [file 12859_2016_1060_MOESM6_ESM.pdf]

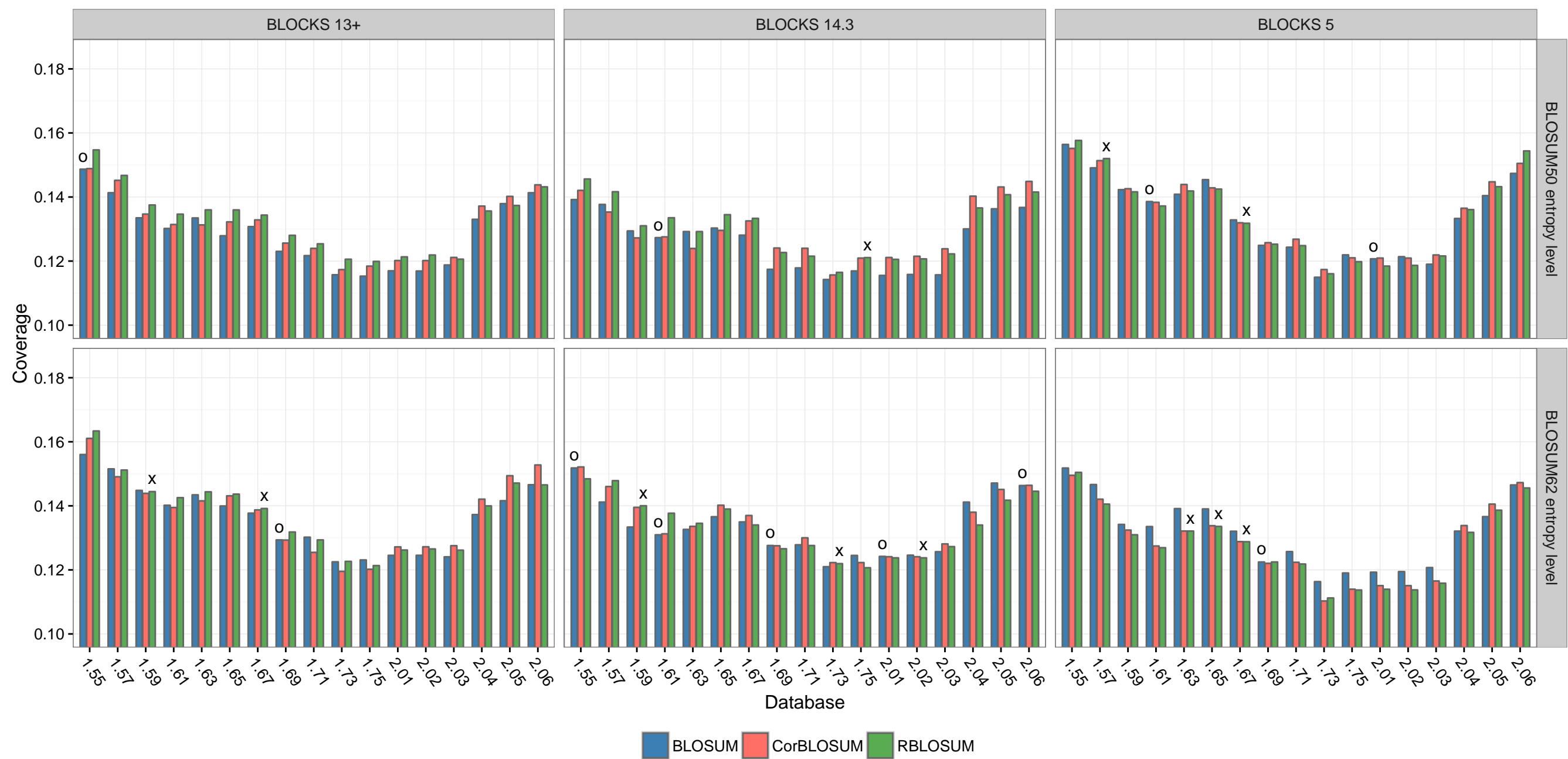

Supplement: Additional file 7 — Figure S5. Progression of the maximum achieved coverage of CorBLOSUM-, RBLOSUM- and BLOSUM-type matrices for all ASTRAL20 test databases. The upper row shows the results for the respective BLOSUM50 entropy level, the lower row for BLOSUM62 entropy level. An insignificant coverage difference between CorBLOSUM and BLOSUM is indicated by an O and between CorBLOSUM and RBLOSUM by an X. The corresponding gap parameter settings are listed in Additional file 2. The BLOSUM62 entropy level substitution matrices derived from BLOCKS 13+ and BLOCKS 14.3 consistently achieved higher coverages than those on the BLOSUM50 entropy level. For the BLOSUM50 entropy level, CorBLOSUM-type matrices performed at least as good as their BLOSUM counterparts in ∼80 % of all tested scenarios and in ∼53 % showed a similar or better performance than the RBLOSUM-type matrices. For the BLOSUM62 entropy level CorBLOSUM matrices showed equally as good or better performance than BLOSUM in ∼49 % while improving performance over RBLOSUM in ∼70 % of all analyzed ASTRAL20 scenarios. (PDF 9.69 kb) [file 12859_2016_1060_MOESM7_ESM.pdf]

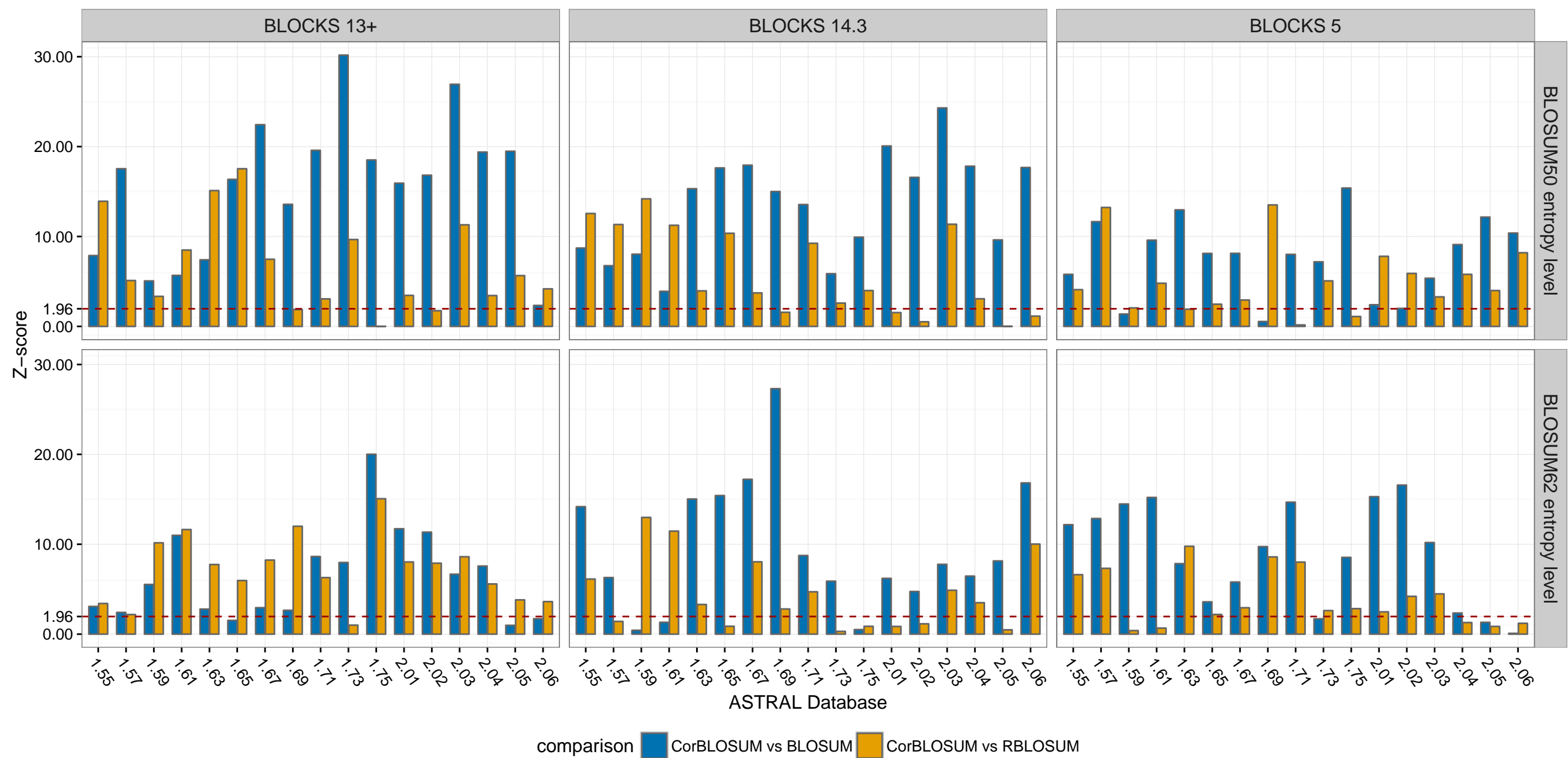

Supplement: Additional file 8 — Figure S6. Z-scores for the coverage comparison of CorBLOSUM with BLOSUM and RBLOSUM based on Bayesian bootstrap for the ASTRAL40 datasets. (PDF 8.61 kb) [file 12859_2016_1060_MOESM8_ESM.pdf]

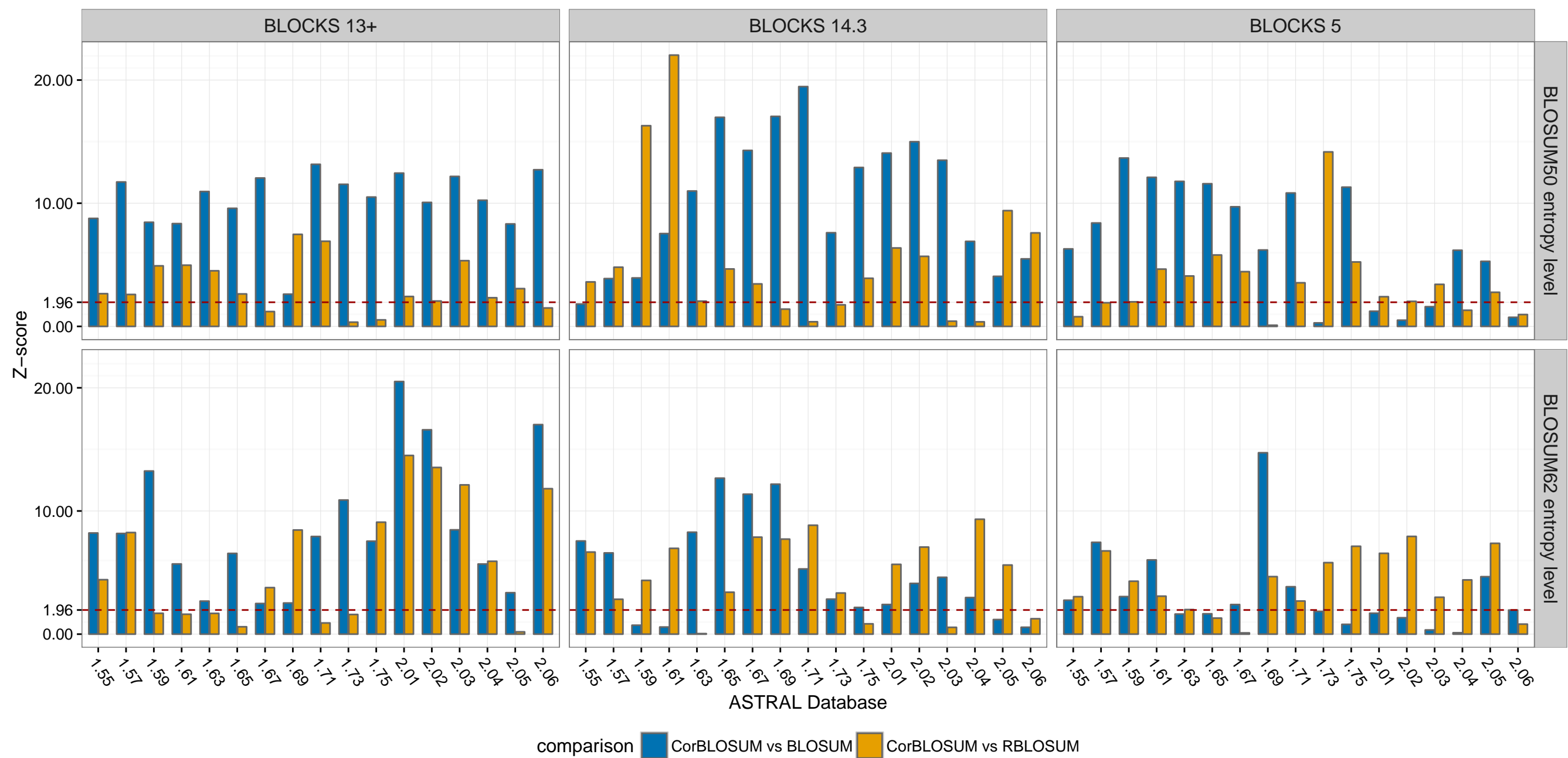

Supplement: Additional file 9 — Figure S7. Z-scores for the coverage comparison of CorBLOSUM with BLOSUM and RBLOSUM based on Bayesian bootstrap for the ASTRAL70 datasets. (PDF 8.55 kb) [file 12859_2016_1060_MOESM9_ESM.pdf]

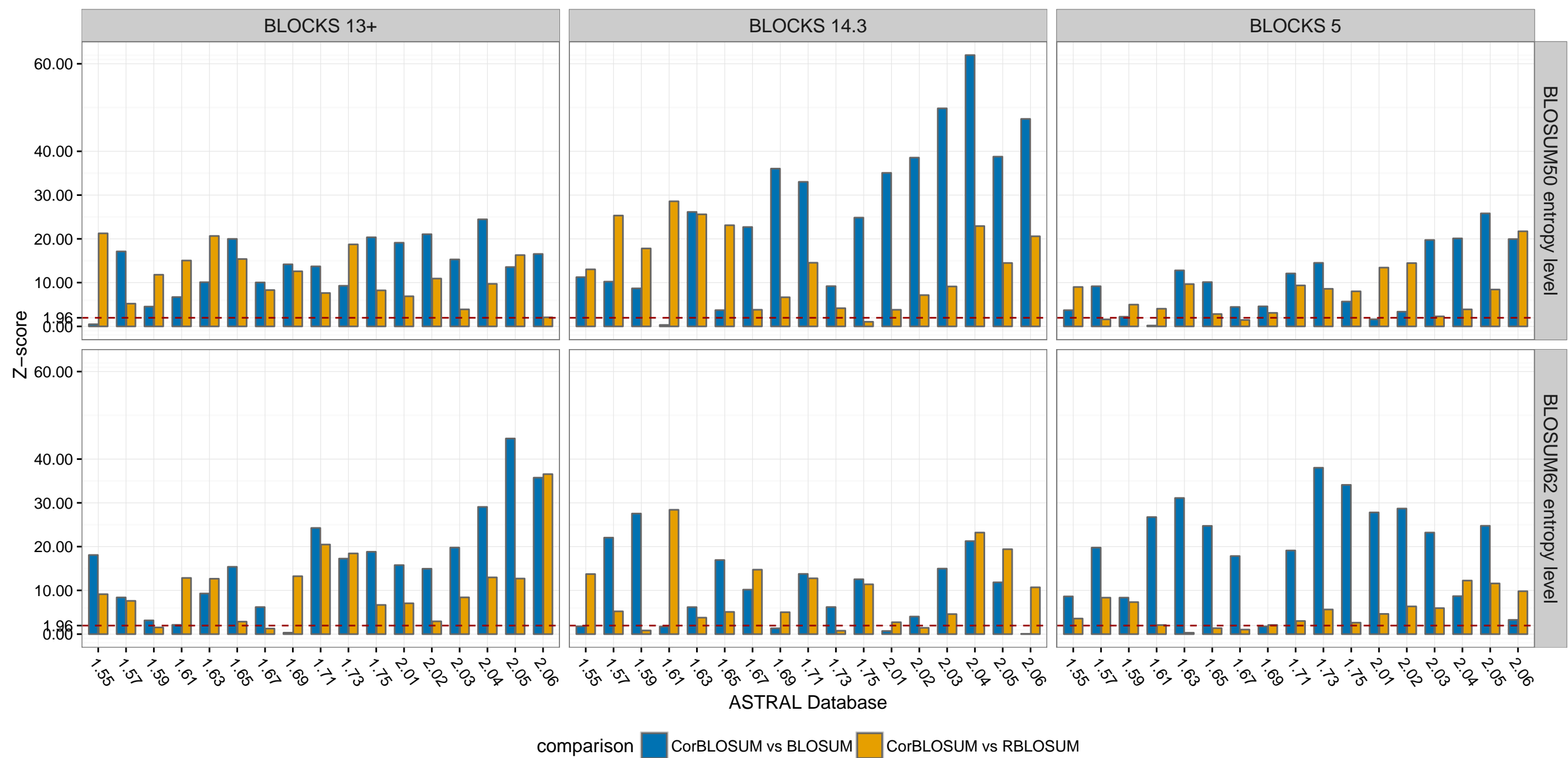

Supplement: Additional file 10 — Figure S8. Z-scores for the coverage comparison of CorBLOSUM with BLOSUM and RBLOSUM based on Bayesian bootstrap for the ASTRAL20 datasets. (PDF 8.91 kb) [file 12859_2016_1060_MOESM10_ESM.pdf]
